# Binding-based proteomic profiling and the fatty acid amides

**DOI:** 10.15761/TR.1000120

**Published:** 2018-11-12

**Authors:** David J Merkler, James W Leahy

**Affiliations:** 1 Department of Chemistry, University of South Florida, Tampa, FL, USA; 2 Center for Drug Discovery and Innovation, University of South Florida, Tampa, FL USA; 3 Department of Molecular Medicine, Morsani College of Medicine, University of South Florida, Tampa, FL USA

## Abstract

Fatty acid amides represent a diverse and underappreciated family of lipids found in vertebrates and invertebrates. The most recognized, most studied, and best understood members of the fatty acid amide family are *N*-arachidonoylethanolamine (anandamide) and oleamide. Over 70 other fatty acid amides have been identified from biological systems and these non-anandamide and non-oleamide fatty acid amides are not well understood: their cellular functions, transport, biosynthesis, and degradation are, at best, partially elucidated. Most of the fatty acid amides are “orphan” ligands for “orphan” or unknown receptors. Interest in the fatty acid amides will wane without a more complete understanding of their function *in vivo* and most of these lipids will be mentioned in a few sentences in reviews on ananamide and/or olemide. In this commentary, we suggest that one strategy to dramatically increase our understanding of any member of the fatty acid amide family is the design, synthesis, and proper use of binding-based profiling probes (BBPPs) based on the structure of a specific fatty acid amide. A BBPP is an analog of a fatty acid amide that enables the controlled covalent attachment of the probe to a fatty acid amide-binding protein and, also, possesses a chemical moiety that will allow the purification and/or detection of the BBPP-labeled proteins. The identification of the proteins that specifically bind a fatty acid amide will foster a better understanding of the function, transport, and metabolism of a fatty acid amide.

## The discovery of the fatty acid amides in biological systems

**Figure 1.**
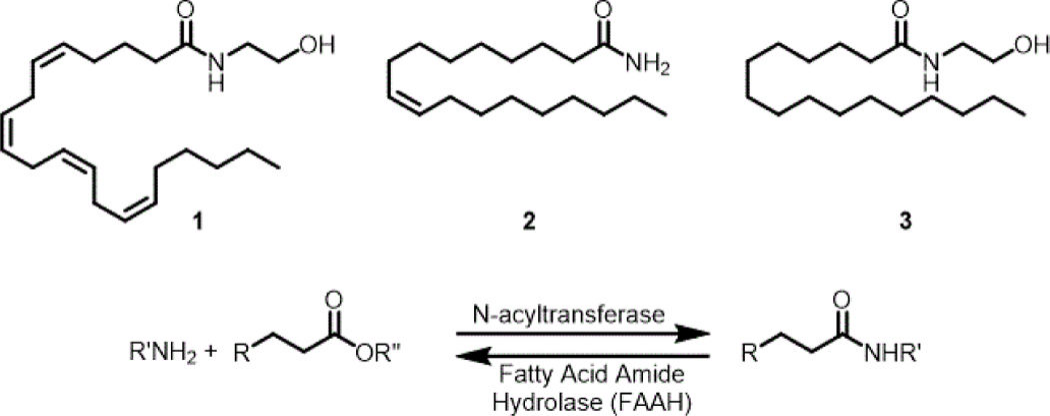
Earliest known fatty acids amides and their biosynthesis/catabolism

The biological occurrence of a fatty acid amide traces back to the 1880’s with the discovery of sphingomyelin by Thudichum [1] and its subsequent structural characterization by Levene in 1916 [2]. The identification of *N*-palmitoylethanolamine (3, Figure 1) from egg yolk [3] and a set of five long chain fatty acid primary amides from human plasma [4] that included both saturated and unsaturated fatty acid components showed that the fatty acid amides were found in biological systems as “stand alone” entities and not only as a component of more complex lipids like the ceramides. Interest in the fatty acid amides increased dramatically with the recognition that *N*-arachidonoylethanolamine (anandamide, 1) is the endogenous ligand to the cannabinoid receptor type 1 (CB_1_) [5] and that oleamide (2) was a regulator of the sleep/wake cycle [6].

The synthesis and degradation of fatty acid amides is not a fully understood process. A number of *N*-acyltransferases have been identified that contribute to the biosynthesis of these compounds, and fatty acid amide hydrolase (which contributes to their hydrolysis) has emerged as a potential therapeutic target. Given the number of these enzymes and their various substrate specificities, it seems clear that the regulation of these compounds is of biological importance. It is therefore surprising that there have been few reported efforts on elucidating the biological targets for these well-regulated compounds. One of the very few that have received attention are the cannabinoid receptors.

## The fatty acid amidome

**Figure 2.**
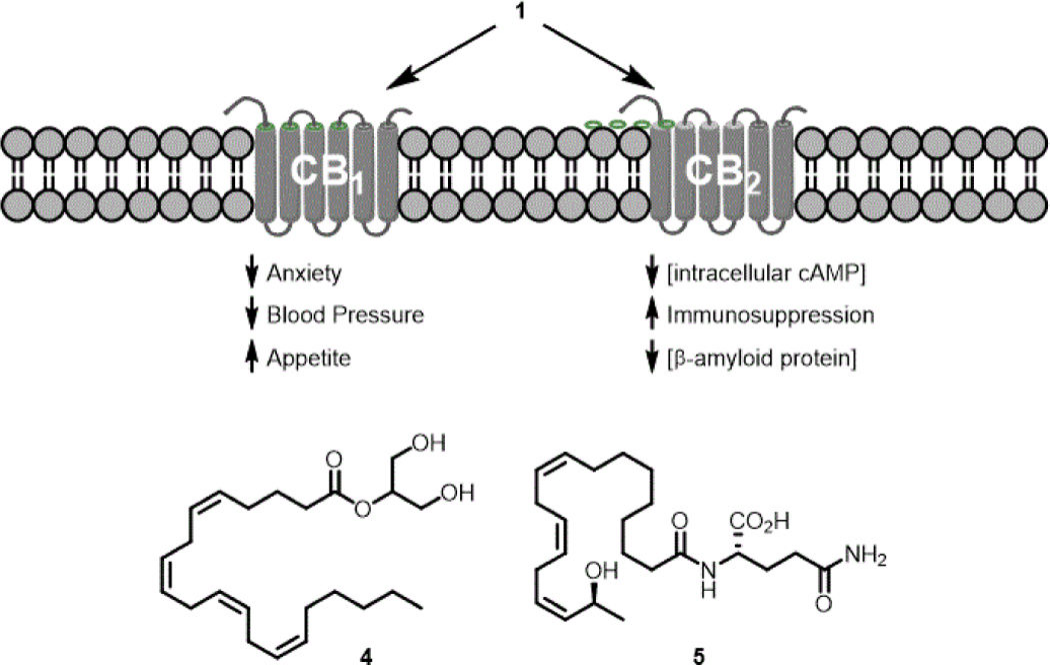
Endocannabinoid regulation and other members of the fatty acid amidome

Endocannabinoids are the family of endogenous molecules that bind to the CB_1_ and CB_2_ receptors; the first identified member of this family was 1. The entire family of endocannabinoids found in a biological system is referred to as the endocannabinoidome. Not all endocannabinoids are fatty acid amides; one important endocannbinoid is 2- arachidonoylglycerol (4, Figure 2) [7]. It is probable some of the fatty acid amides are not endocannabinoids. Thus, the fatty acid amidome is a better term to describe the family of fatty acid amides found in a biological system. Numerous studies of the fatty acid amidome from vertebrates and invertebrates have been reported and the family of known biologically-occurring fatty acid amides is >70, including the *N*-fatty acylethanolamines, *N*-fatty acyldopamines, *N*-fatty acylserotonins, long-chain fatty acid primary amides, and the lipoamino acids [8–10].

Hundreds of different fatty acid amides are possible given the diversity of fatty acids and biogenic amines found in living systems. Oxidation/hydroxylation of the fatty acyl moiety by lipoxygenase or cytochrome P_450_ serves to dramatically increase the potential number of different fatty acid amides [11]. An important example of a fatty acid amide possessing a hydroxylated fatty acyl chain is *N*-(17-hydroxylinolenoyl)-L-glutamine (volicitin. 5, an elicitor of plant volatiles), identified from the oral secretions of the beet armyworm [12].

The fatty acid amidome is actually a subset of a larger family of *N*-acylamines, the amidome. The *N*-myristoylation [13], *N*-palmitoylation [13], and *N*-acetylation [14,15] of proteins and peptides have been described, a family of *N*-succinoylated amines have been identified in *C. elegans* [16], the *N*-acetylation of amines is critical in xenobiotic­ detoxification [17], the regulation of the urea cycle in mammals [18], and in the sclerotization of the cuticle in insects [19]. In short, the *N*-acylamines class of compounds, R-CO-NH-R’, are ubiquitous and clearly critical to life.

## Fatty acid amides: possibilities and challenges

**Figure 3.**
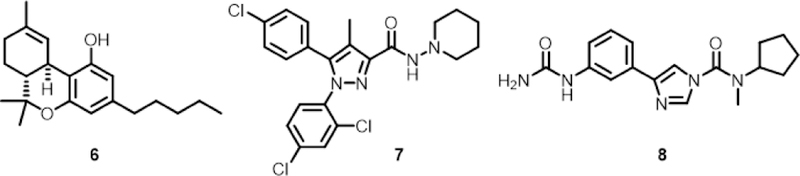
Exogenous cannabinoid ligands

∆^9^-Tetahydrocannabinol (THC, 6, Figure 3) is the primary compound responsible for the psychoactive effects of marijuana [20] and, like anandamide, THC binds to the CB_1_ receptor with high affinity, K_d,CB1-anandamide_ = 80 nM and K_d,CB1-THC_ = 40 nM [21]. The strong ties of anandamide, THC, and the cannabinoid receptors to human health are at the center the “medical marijuana” controversy, but also are an opportunity for the development of endocannabinoid system-based drugs to a variety of human diseases, including chronic pain, anxiety, depression,­ Parkinson’s disease,­ schizophrenia, obesity, and substance abuse. A list of clinically tested, endocannabinoid drugs is included in a recent review by Di Marzo [10]. Two examples from this list are rimonabant (7) and BIA10–2474 (8). Rimonabant, a selective CB_1_ reverse agonist, was approved for use in Europe in 2006 to treat obesity, drug addiction, and smoking [22]. Approval for rimonabant was rescinded in 2009 due to serious side effects: severe depression and suicidal behavior [23,24]. BIA 10–2474, an irreversible inhibitor of fatty acid amide hydrolase, was in Phase 1 clinical trials for the treatment of neuropathic pain, anxiety, and other CNS indications based on its ability to increase the cellular levels of anandamide [10,25]. The Phase 1 trials of 8 were a disaster resulting in the death of one volunteer and long-lasting, mild-to-severe neurological symptoms in four others [26] The complexity of the endocannabinoid system coupled with undesirable outcomes when 7 and 8 were used clinically highlight the challenges in developing drugs targeting the endocannabinoid system. These challenges can be viewed as either a deterrent or an opportunity to future efforts in developing endocannabinoid system-targeted drugs.

We view the challenges as an opportunity – an opportunity that can only be exploited by a deeper understanding of the endocannabinoid system coupled to a deeper understanding of the structurally-related fatty acid amide system. One underappreciated issue in considering drugs targeting the endocannabinoid system are how such drugs could alter the fatty acid amidome and/or bind to off-target fatty acid amide-binding proteins. Since FAAH is likely involved in the hydrolytic degradation for most of the fatty acid amides [27], inhibition of FAAH would increase the cellular levels of not only anandamide, but also other fatty acid amides. Furthermore, it is certainly plausible that a drug designed to bind to CB_1_ or CB_2_ could, also, bind to another fatty acid amide-binding protein because of the structural similarity between the different classes of the fatty acid amides. We [28] and others [8,10] have pointed out that little is known about the proteins that bind most of the fatty acid amides: receptors, transporters, enzymes involved in biosynthesis and degradation, and proteins allosterically regulated by individual fatty acid amides.

## The design and implementation of binding-based pro-filing probes targeted against specific fatty acid amides

Developed by the Cravatt group [29], activity-based proteomic profiling (ABPP) is a superb technology to identify proteins that bind to a ligand. Arguably, the most important aspect to the proper use of ABPP technology is the design of the ABPP probe – an analogue of ligand that possesses a reactive group to attach the probe to proteins that bound the ligand and a group that enabled the purification and/or purification of probe labeled proteins. ABPP has been used to identify novel enzymes and receptors [30]; selective, tight-binding inhibitors [31]; off-targets for drugs [32]; new metabolic pathways [30]; and even novel substrates for known enzymes [33].

**Figure 4.**
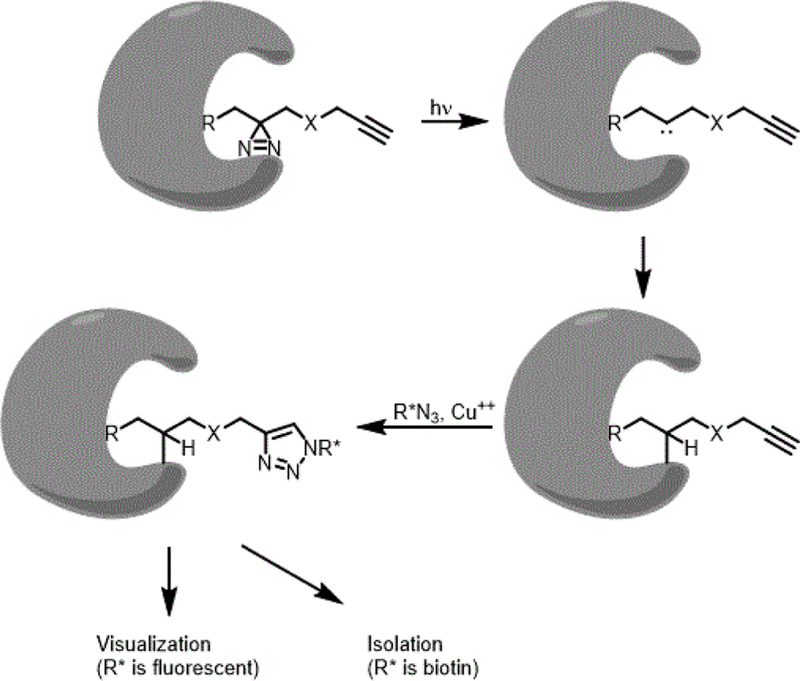
Schematic of covalently attaching and utilizing an ABPP

ABPP probes were first designed to label a reactive amino acid with the active site of mechanistically related enzymes. However, the ABPP approach does not work well with proteins that do not contain a reactive amino acid in the probe binding site: receptors, transporters, or a protein that binds the probe at an allosteric site. One solution to this limitation is to construct a binding-based proteomic profiling (BBPP) probe that incorporates a photoactivatable moiety that provides a controllable method to attach the probe into a chemically inert probe-binding site (Figure 4). The construction of BBPP probes that possess a diaziridine or azido functionality are obvious choices. Upon UV irradiation, diazirines form carbenes and azides form nitrenes; reactive moieties that can insert into C-H and N-H bonds to covalently (and thus irreversibly) decorate an unreactive binding site with the probe. BBPP probe-labeled proteins can be visualized and/or purified by either incorporating a biotin or fluorescent group directly into the probe or incorporating an alkyne moiety into the probe to “click-in” [34] a biotin or fluorescent group (R*) into the probe after the probe-labeled proteins have been produced. We have designed BBPP probes based on 8-azido-adenosine analogues to profile adenosine/adenylated binding proteins in mouse neuroblastoma N_18_TG_2_ cells [35] and Niphakis *et al*. [36] generated diazirine-containing analogs of anandamide to profile anandamide-binding proteins in HEK293T cells. One intriguing result from Niphakis *et al*. [36] was that anandamide specifically binds to nucleobindin-1 (NUCB1). NUCB1 is found in the Golgi and was thought to bind Ca(II) and DNA [37] and was not known to bind lipids. Data presented by Niphakis *et al*. [37] indicate that NUCB1 may function *in vivo* to transport fatty acid amides to FAAH for degradation. This result demonstrates the power and utility of the BBPP strategy to identify novel lipid binding proteins. BBPP probes like these discussed here have been designed to profile proteins that bind other lipids, including the ceramides [38], sphingosine [39], diacylglycerols [40], and Lipid II [41]. In fact, Bockelmann *et al*. [38] used their ceramide-based BBPP probe to identify a novel ceramide-binding protein.

BBPP probes targeted against any fatty acid amide can be readily synthesized, methods to use the probes to profile soluble and membrane-bound proteomes have been described, and protocols to validate the probe-labeled proteins as true lipid-of-interest-binding proteins have been described. It is time to apply the power of BBP to the fatty acid amides to foster a better understanding of their function and to define the proteins to which these binds. The identification of protein binding partners for specific fatty acid amides will foster a greater understanding of their function, of their biosynthesis and degradation, will identify new targets for the development novel human therapeutics and may reveal off-target proteins for drugs targeted against the endocannabinoid system.
